# ZnO nanostructured materials for emerging solar cell applications

**DOI:** 10.1039/d0ra07689a

**Published:** 2020-11-24

**Authors:** Arie Wibowo, Maradhana Agung Marsudi, Muhamad Ikhlasul Amal, Muhammad Bagas Ananda, Ruth Stephanie, Husaini Ardy, Lina Jaya Diguna

**Affiliations:** Material Science and Engineering Research Group, Faculty of Mechanical and Aerospace Engineering, Institut Teknologi Bandung Jl. Ganesha 10 Bandung 40132 Indonesia ariewibowo@material.itb.ac.id; Research Center for Nanoscience and Nanotechnology, Institut Teknologi Bandung Jl. Ganesha 10 Bandung 40132 Indonesia; Research Center for Metallurgy and Materials, The Indonesian Institute of Sciences Puspitek Serpong Banten 15314 Indonesia; Department of Renewable Energy Engineering, Universitas Prasetiya Mulya Kavling Edutown I.1, Jl. BSD Raya Utama, BSD City Tangerang 15339 Indonesia lina.diguna@prasetiyamulya.ac.id

## Abstract

Zinc oxide (ZnO) has been considered as one of the potential materials in solar cell applications, owing to its relatively high conductivity, electron mobility, stability against photo-corrosion and availability at low-cost. Different structures of ZnO materials have been engineered at the nanoscale, and then applied on the conducting substrate as a photoanode. On the other hand, the ZnO nanomaterials directly grown on the substrate have been attractive due to their unique electron pathways, which suppress the influence of surface states typically found in the former case. Herein, we review the recent progress of ZnO nanostructured materials in emerging solar cell applications, such as sensitized and heterojunction architectures, including those embedded with promising perovskite materials. The remarkable advancement in each solar cell architecture is highlighted towards achieving high power conversion efficiency and operational stability. We also discuss the foremost bottleneck for further improvements and the future outlook for large-scale practical applications.

## Abstract 2

Zinc oxide (ZnO) has been considered as one of the potential materials in solar cell applications, owing to its relatively high conductivity, electron mobility, stability against photo-corrosion and availability at low-cost.

## Graphical abstract

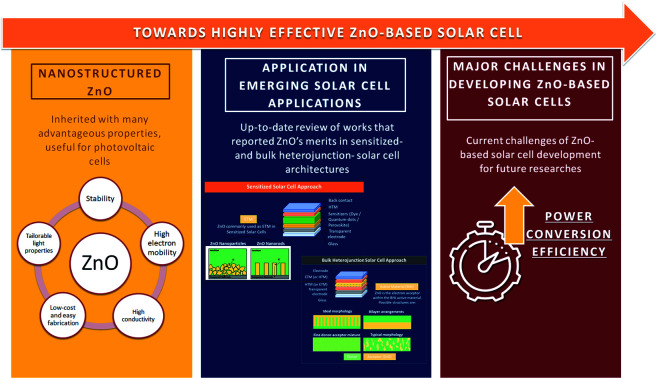

## 1 Introduction

Emerging solar cell technologies that use complex and advanced materials, such as perovskite, dye-sensitized, organic, quantum dot and multijunction, were born to answer the challenges for conversion efficiency and durability. None of the developed solar cell technology has closely achieved the theoretical energy conversion limit to 90%. The primary cause of the inefficiency of solar cells is related to the energy bandgap, as well as the transmission and thermalization losses. This is strongly related to the properties of the active material, including their defects. In addition, the intrinsic stability of these materials affects the lifetime or durability of the solar cells systems. The desired properties of the charge transport materials for solar cells application are ideal energy levels that correspond to the high absorption efficiency of the solar spectrum, high carrier mobility, good conductivity, and efficient extraction of the excited carriers.

**Fig. 1:**
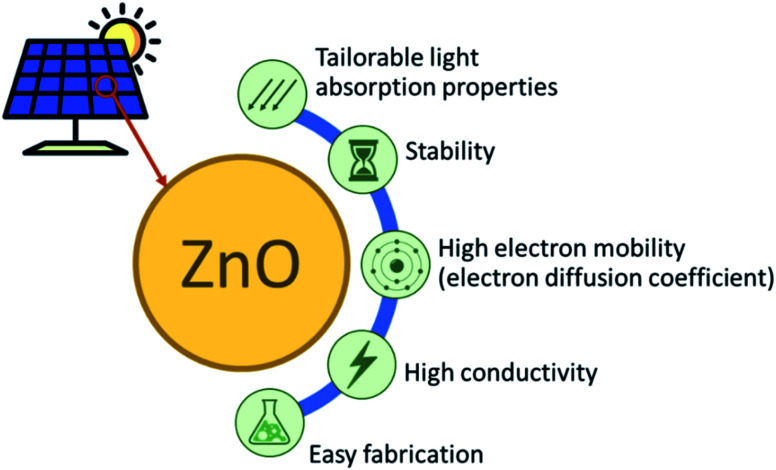
The merits of ZnO for solar cell application.

ZnO materials, one of the group II–VI binary compound semiconductors, have been considered in solar cell applications due to their stability, high conductivity, high electron affinity and excellent electron mobility. Fig. 1 illustrates the advantages of ZnO as an active material for solar cell applications. ZnO materials are wide bandgap semiconductors with a band gap of 3.1–3.3 eV that absorb light only in the UV region. ZnO can also be coupled with smaller energy gap materials, such as dye sensitizers, organic polymers, and smaller band gap semiconductors, to extend their light absorption to the visible region. The bulk ZnO has been reported to have an exciton Bohr radius (*a*_B_) of 2.34 nm.^1^ This is comparable to the significant confinement effects, experimentally observed for the solution phase synthesized ZnO particles with the particle radii of less than about 4 nm, due to the relatively small effective masses for ZnO, *i.e.*, *m*_e_ = 0.26*m*_0_, *m*_h_ = 0.59*m*_0_ and *m*_0_ is the free electron mass.^2^ The high electron mobility of ZnO makes this material attractive for solar cell application, 205–300 for bulk^3,4^ and 1000 cm^2^ V^−1^ s^−1^ for nanorod ZnO.^5^ These values are relatively high compared to those of the commonly used TiO_2_, *i.e.*, 0.1^−4^ cm^2^ V^−1^ s^−1^.^6^ Moreover, the electron diffusion coefficient is 5.2 for bulk and 1.7 × 10^−4^ cm^2^ s^−1^ for the nanoparticulate film ZnO.^7^ Conversely, in the bulk and nanoparticulate film TiO_2_, the electron diffusion coefficient becomes 0.5 and 10^−8^−10^−4^ cm^2^ s^−1^, respectively.^8^ ZnO is also well-known as a polymorph, having a different type of structure depending on the synthesis method. The nanomorphology of ZnO comprise nanospheres, nanowires, nanorods, nanoflower, nanotubes, nanocrystals, and 3D nanostructures (core–shell). These excellent attributes have made ZnO widely applied in many areas, such as sensors, surface coating, porous ceramics, photodetectors, nano-piezoelectric, and supercapacitors, in addition to solar cells. There are also many available methods to prepare ZnO nanomaterials from different pathways (biological/physical/chemical), such as green synthesis using microorganisms, hydrothermal, sol–gel, electrochemistry, inkjet printing, atomic layer deposition, and sputtering technique.

**Fig. 2:**
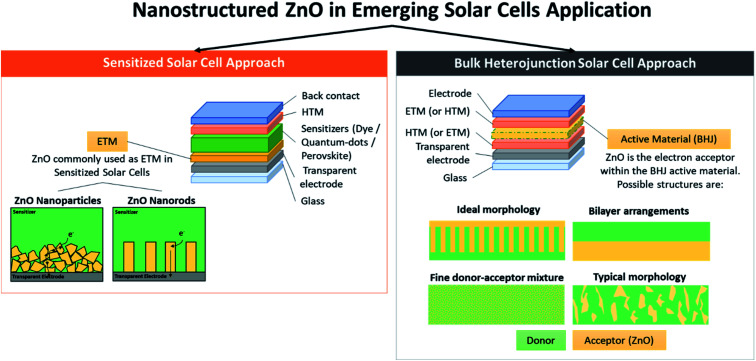
Nanostructured ZnO in emerging solar cell applications.

In this review, the latest application of ZnO in third-generation solar cell technologies is thoroughly discussed. Different solar cell architectures, *i.e.*, sensitized solar cells and heterojunctions solar cells, including those embedded with promising perovskite materials, will be reviewed in accordance with the influence of the synthesis strategies on the ZnO properties. These approaches include the application of different nanostructures of ZnO, deposition and post-treatment method, and inclusion of dopant materials (Fig. 2). The discussion will be concluded by the proposal of future strategies to improve the current achievements Fig. 2.

## 2 Emerging solar cell application

### 2.1 Sensitized solar cells

Dye-sensitized solar cells (DSSCs) were first proposed by O'Regan and Grätzel in 1991, and have attracted great interest as an alternative to conventional silicon solar cells. The fabrication is straightforward and low-cost, with good long-term stability and energy conversion efficiency exceeding 10%.^9^ Ru-based dyes are utilized as photosensitizers that are attached to the mesoporous metal oxide photoelectrode with large surface areas, and absorb solar energy efficiently. The electrons injected by the optically excited dye into the metal oxide conduction band diffuse across the semiconductor film layer, and reach the back contact. Redox couples diffuse in solution, which are in turn reduced at the counter electrode, and regenerate the oxidized dye. Because of the sensitizers, the high expense to provide the typical dyes (N3, N719) has encouraged the alternative use of narrow band gap semiconductor quantum dots (QDs), owing to their size-tunable optical properties to match the solar spectrum. Moreover, QD-sensitized solar cells (QDSCs) have the capability of producing multiple electron–hole pairs per photon quantum yields greater than 1 through impact ionization.^10–12^ In the early studies, QD-sensitized solar cells showed performances well below the expectation, where the achieved efficiency in two-electrode configurations was below 1%.^13,14^ Later, the efficiency of 2.7% reported by Diguna *et al.* in 2007 brought about a resurgence in the development of QDSCs.^15^ The different morphologies of the photoanode have also been considered to enhance the light-harvesting efficiency, such as the inverse opal due to its large interconnected pores for better penetration of the dye and photon confinement at a wavelength near the photonic band gap for the significant enhancement of dye absorption.^16^ In the following sections, the application of various ZnO-nanostructured materials and their recent progress in DSSCs, PSSCs, and QDSCs are discussed in detail.

#### 2.1.1 Dye-sensitized solar cells

In 1980, Matsumura *et al.* reported 2.5% energy conversion efficiency under monochromatic light at 562 nm using ZnO porous disks sensitized with rose bengal.^17^ The efficiencies for the nanoporous ZnO thin film were then reported to reach as high as 2% under 56 mW cm^−2^ illumination with the ruthenium-complex dye,^18^ and 2.5% under 99 mW cm^−2^ illumination with the mercurochrome sensitizer.^19^ The mercurochrome dye was bonded to the ZnO surface in a way that is similar to TiO_2_ through the carboxylate linkage. On the other hand, the different adsorption of the ruthenium-based dye caused the typically poor performance of ZnO compared to TiO_2_. The formed agglomerates of Zn^2+^ and the ruthenium dye molecules during dye adsorption were reported to fill up the nanopores of the ZnO electrode, and act as an insulating layer blocking the injected electrons from the dye molecules to the semiconductor.^20,21^ The isoelectric point of ZnO is ∼9 and that of TiO_2_ is ∼6, implying that ZnO is more fundamental than TiO_2_. Therefore, the ZnO electrode has poor chemical stability in the presence of an acidic dye.^22^ The deprotonation of the carboxyl groups of the ruthenium dye made the dye solution relatively acidic, and can etch ZnO surfaces during the dye adsorption. The dissolution of ZnO by the acidic carboxylic groups of the dyes takes place at the crystal surface. This leads to the formation of Zn^2+^/dye complexes, and thus prevents the efficient electron injection. A nanoporous ZnO electrode sensitized with ruthenium bipyridyl complex has been reported with an efficiency of up to 5%. This was achieved by suppressing dye aggregation and Zn^2+^/dye complex formation with the addition of basic KOH to the dye solution, and by applying pressure and excluding organic additives in the ZnO film preparation for better interfacial kinetics.^23,24^ The photoelectrochemical characteristics of the ZnO electrode strongly depended on the electrode morphology, such as the size, the shape of the particles and the porosity, whereas the 8 mm thick ZnO film consisting of 150 nm spherical particles was found to exhibit efficient electron transport in the nanostructured electrode with small recombination losses.^25^

The traditional nanoparticle film in DSSCs effectively provided a large surface area for the adsorption of light-harvesting dye molecules. However, the electron transport through the nanoparticle film was based on trap-limited diffusion, a slow mechanism that limited the device efficiency, especially at longer wavelengths. Therefore, an array of oriented 1D nanostructures, such as nanowires, nanorods and nanotubes, was later introduced as a promising solution to increase the electron diffusion length in the anode. The aspect ratio (length divided by diameter) of the nanowires may be up to 1000, while that of the nanorods is much smaller, usually less than ten depending on the synthesis method and condition. In 2005, the ZnO nanowire photoanode with an array length between 20–25 μm and a surface area of up to one-fifth of a nanoparticle film was synthesized using a seeded growth process. The ZnO quantum dots as a 10–15 nm thick film was initially deposited onto the FTO substrate by dip coating. Later, wires were grown from these nuclei through thermal decomposition of the zinc complex. With the sensitization of the ruthenium dye, it demonstrated 1.5% efficiency at one sun.^26^ In the same studies, the electron injection from the photoexcited ruthenium dyes into nanowires and nanoparticles was evaluated by using femtosecond transient absorption spectroscopy. The resulting bi-exponential kinetics of the nanowires (time constants of <250 fs and around 3 ps) confirmed the faster electron injection relative to the nanoparticles with a tri-exponential response (time constants of <250 fs, 20 ps and 200 ps). The Core-shell concept was then applied to the ZnO nanowire photoanode by coating ZnO nanowires with the thin shell of amorphous Al_2_O_3_ or anatase TiO_2_ by atomic deposition.^27^ A very thin alumina shell acted as a tunnel barrier that improved the *V*_oc_ by impeding recombination, but blocked electron injection as it became thicker. On the contrary, titania shells were found to suppress the rate of recombination and improve the open-circuit voltage and fill factor, in which the shell thickness of 10–25 nm caused a dramatic increase in efficiency by up to 2.25% under one sun.

The photovoltaic properties of the ZnO nanorods were reported to be dependent on not only the rod size, but also on their orientation.^28^ Vertically aligned ZnO nanorods with N179 sensitization exhibited very low power conversion efficiency, *i.e.*, 0.22% and 0.09% for hydrothermally grown and vapor deposited ∼3.5 μm-length ZnO nanorods.^29^ Modification on the ZnO nanorod surface with gold nanoparticles formed a Schottky barrier, and then blocked the electron transfer back from ZnO to the N719 dye and electrolyte, thus increasing the efficiency up to 1.2%.^30^ Doping the ZnO nanorods with Al also improved the performance of the N719-sensitized ZnO nanorods from 0.05% to 1.34%.^31^ Here, the occupation of the trivalent Al^3+^ in the divalent Zn^2+^ ion site increased the electron concentration and thus the electrical conductivity, allowing electrons to move easily into the Al-doped ZnO conduction band. Chemically etching the center part of the electrochemically deposited ZnO nanorods produced a ZnO nanotube array. The employment of the 5.1 μm-length ZnO nanotube as a photoanode with N719 sensitization showed an efficiency of 1.18%.^32^ On the other hand, ZnO nanotube photoanodes templated by an anodic aluminium oxide exhibited an efficiency of 1.6% under AM 1.5 illumination.^33^ Combined with atomic layer deposition to conformally coat the nanotube pores, the design could provide a direct path for charge collection over tens of micrometers thickness, indicated by the exceptional photovoltage of 739 mV and fill factors of 0.64. Unfortunately, the low photocurrent caused by insufficient light harvesting due to the small roughness factor and photoanode reflectivity/scattering (light coming from the counter electrode side) limited the overall efficiency. The increase of the surface area was required to further improve the energy conversion efficiency.

The dye in DSSCs also plays a critical role in enhancing the PCE. A compatible dye in the ZnO-based DSSCs will improve the electron injection efficiency. The enhancement of the electron injection efficiency depends on the electronic coupling and relative energy levels between the dye and the semiconductor, the lifetime of the dye, and ultimately on the density of the electron-accepting state (DOS) in the semiconductor. The electronic coupling between the dye and semiconductor relies on the selection of the dye group for the appropriate semiconductor. The dyes shall be identified or synthesized, for which the LUMO (Lowest Unoccupied Molecular Orbital) and HOMO (Highest Occupied Molecular Orbital) levels match, respectively, with the conduction band energy levels and the valence band energy levels of the semiconductor. The lifetime of the excited state is a fundamental property of the photosensitizer. The dye with longer life in the excited state is expected to be easier for charge transfer.^34^

In contrast to TiO_2_, ZnO is not compatible with the Ru dye due to its more alkaline nature, making it susceptible to low pH conditions. The Ru dye also removes Zn^2+^ ions from the ZnO lattice. The improper immersion duration of the ZnO film in the ruthenium dye could lead to a lower efficiency for the DSSC due to ZnO film release, reduction in the number of free electrons, promotion of the recombination process, and transformation of the porous film to a denser one.^34,35^ In some recently reported research studies (Table 1), metal-free dyes such as indoline dyes, D205 and D149,^36^ heptamethine-cyanine dye (KFH-3),^37^ C220,^38^ carbazole dyes,^39^ anthocyanins,^40^ and Xanthenes^41^ were found to be the right-choice for this purpose. Recent studies by Chang *et al.* and Lin *et al.* have shown that dyes with relatively lower acidity (indoline dye coded D149) show a relatively good compatibility with ZnO. Both types of research studies conducted found that an efficiency higher than 5% can be achieved for the flexible ZnO-based DSSC.^42,43^

**Table 1:** The effect of the ZnO nanostructure, dye type, and doping type on the photovoltaic performance of ZnO based-DSSCs

ZnO nanostructure	Dye	Doping	PCE (%)	Ref.
Nanorods	Crystal violet	La	0.36	^49^
Nanoflower	N719	Li	1.23	^48^
Nanoparticles	Mercurochrome	Ag	2.02	^47^
Nanospheres	N719	In	2.7	^51^
Hollow spheres	N719	—	3.28	^53^
Nanorods	N719	—	3.75	^54^
Nanoparticles	N719	Iodine	4.01	^45^
	D205		4.44	
Pomegranate	N719	—	4.35	^53^
Nanoparticles	CYC-B1	—	5.4	^55^
Nanosheets	N3	B	6.75	^46^

In a more recent study, several efficient metal-free organic sensitizers were developed by Selopal *et al.* called B18, BTD-R, and CPTD-R for ZnO-based DSSCs. The B18 dye provides better photovoltaic properties than the other two dyes in the hierarchically structured ZnO and commercial TiO_2_ due to the higher electron injection potential and better light harvesting. The TCD/TVD results showed that the device with dye B18 had a better *τR* and negligible shift in dye-sensitive photoanode CB compared to the CPTD-R and BTD-R dye-based devices.^44^

Another way to improve electron transport in the Zn photoanode, which also enhances cell efficiency, is to add dopants to the ZnO film. Dopants will fill the holes in the cell, thus increasing the recombination injected electron in the semiconductor.^34^ Several materials such as iodine,^45^ B,^46^ Ag,^47^ Li,^48^ La,^49^ Sr,^50^ In,^51^ and Nd^52^ could be doped into the semiconductor film of ZnO, according to some reports. Zhao *et al.* used iodine-doped ZnO-based as a photoanode in dye-sensitized solar cells with indoline D205 and N719 as the sensitizers. The result demonstrated that iodine-doping boosts the efficiencies compared to the cells without iodine. The efficiencies of the D205-I-ZnO based DSSC and N719-I-ZnO based DSSC were enhanced by 20.3% and 17.9%, respectively.^45^ Mahmood and Park reported a cell fabricated with ZnO nanosheets doped with boron and using the N3 dye as a sensitizer, achieving a significant enhancement in PCE (6.75%) in contrast to the undoped ZnO nanosheets (2.62%) and BZO films only including nanosized crystallites (3%).^46^ The work from Lanjewar *et al.* revealed that upon doping with Ag, the band gap is sharply reduced and the resulting Ag:ZnO photoelectrode could absorb the visible light range to a great extent. The most massive reduction in the band gap was achieved from 3.28 eV for the pure ZnO film to 2.65 eV for Ag:ZnO with 10.3 wt% doping with the enhancement of PCE from 0.55% to 2.02%.^47^ In a very recent study, Aksoy and collaborator investigated the addition of Li into ZnO powder using N719 dye for dye-sensitized solar cells. The result suggested that the nanoflower morphology was formed, and the efficiency of the cells rise to the value of 1.23%. The enhanced efficiency was associated with the change in the morphology, and an improvement in the crystallinity in Li-doped ZnO based DSSCs.^48^ Research conducted by Goel and co-worker demonstrated that the La-doped ZnO-based nanopowder solar cell exhibited superior photovoltaic performance when compared to the pure ZnO-based cell. The light harvesting efficiency (*η*) increased from 0.20% to 0.36% on doping with La.^49^ In a study by Chava *et al.* using Indium-doped ZnO, the maximum photoconversion efficiency of 2.7% was achieved on 0.2 In–ZnO photoelectrode films that consisted of nanosized crystallites and aggregated spheres of nano-crystallites.

The enhancement in the performance of DSSCs with 0.2 In–ZnO films was attributed to the strong light scattering phenomenon of aggregated spheres within the photoelectrode film, the pore size of the aggregates, which offers a more porous structure for dye infiltration and electrolyte diffusion.^51^

#### 2.1.2 Quantum dots-sensitized solar cells

ZnO nanostructured materials have been explored as a photoanode in sensitized solar cells with narrow semiconductor quantum dots as an alternative light absorber that replaces dye molecules. The properties of quantum dots (QDs) and photoanode films and the interconnectivity between them play significant roles in the device performance. The implementation of a particular fabrication method to obtain specific characteristics may lead to some challenging issues. Pre-synthesized ZnO nanomaterials usually are applied to a conductive glass substrate by using squeegee methods.^56^ On the other hand, QDs could be synthesized and later adsorbed on the photoanode or directly *in situ* grown on the photoanode.^12,57,58^ This pre-synthesized method of QD permits greater control over the size, shape and surface properties of QD. However, the resulting QD seems to be a monolayer, delicately adsorbed on the photoanode surface. Thus, appropriate crosslinking molecules are usually required to deposit QDs properly on the photoanode. The deposition of both pre-synthesized ZnO materials and QDs may affect the contact between the semiconductor films and conductive glass, or between the semiconductor films and QDs. The ultrasonic spray pyrolysis method in depositing both ZnO particles and CdS QDs on the conductive glass has been proposed to create a good contact for efficient electron transport from CdS QDs to a conductive glass *via* ZnO photoanode, as shown by the increased short circuit current density.^59^ On the other hand, the *in situ* growth method might also be implemented to grow the ZnO photoanode directly on the substrate^60^ and/or QDs deposition on the ZnO surface.^61^ This method may provide a good contact of the ZnO/substrate interface and more extensive, uniform deposition of QDs in the entire photoanode.

Different ZnO structures have also been pursued to achieve specific features, such as better electron transport than those obtained in nanoparticles. One-dimensional (1D) structures such as nanorods, nanowires, and nanotubes, had vertically aligned electrical pathways and reduced particle-to-particle hopping of electrons usually found in the nanoparticle network, which are expected to increase the efficiencies of those photoelectrical devices. Modification on the ZnO nanorod, *i.e.*, taper-like arrays, could minimize the charge transfer resistance, thus increasing the short current circuit and conversion efficiency consequently for the case of nitrogen-doped graphene QDs.^62^ Moreover, the efficiencies of the ZnO nanorod array devices are limited by their low light-harvesting ability. Nanotubes have a larger surface area than nanorods of similar length and diameter. ZnO nanotube arrays have been proved to have a superior ability as compared with ZnO nanorod arrays due to the excellent light scattering efficiency on account of their 1D tubular nature.^63^ Taking into account the critical role of the QDs interfaces in carrier relaxation,^64^ interfacial engineering (such as surface modification) has reported over one decade in suppressing surface defects, leading to smooth electron transfer and thus improving the photovoltaic performance of the QD-sensitized TiO_2_ solar cells.^15,56,58^ The similar approach has also been introduced on the CdS/CdSe quantum dot co-sensitized ZnO photoanode by MnS passivation layer to suppress charge recombination at the photoelectrode/electrolyte interface, and also enhance the light-harvesting capability in terms of both absorbance intensity and absorption range.^65^ One option to increase the solar cell performance is by structure architecture, enabling the light scattering and thus, the light-harvesting efficiency. ZnO hollow microspheres have been found to generate light scattering and thus improve the power conversion efficiency of the Zn_*x*_Cd_1−*x*_Se QDSSC.^66^ On the other hand, this hollow structure may also lead to the defects located at the surface. To tackle this issue, the TiO_2_ passivation layer on the hollow microsphere surface has been reported recently to improve the CdSe/CdS QDSSC performance. The improvement not only produced better light harvesting, but also reduced the charge recombination and lengthened the electron lifetime.^67^ QDs have a unique capability of producing multiple electron–hole pairs per photon quantum yields greater than 1 through impact ionization.^10,11^ The attachment of QDs on the ZnO surface has been reported to possibly speed up carrier relaxation in the QDs, which is an essential factor for hot-carrier energy harvesting *via* multiple electron generation and hot electron transfer, depending on the exact linker molecules.^68^ Although an external photocurrent quantum efficiency of more than 100% has been reported for the hererojunction ZnO/PbSe QD solar cell,^69^ to the best of our knowledge, there are no reports on QDSSCs yet.

Besides the design of the photoanode in terms of its semiconductor properties, interfaces and crosslinking molecules, the redox couples and counter electrodes used should also be considered carefully for higher photoconversion efficiency to ensure a smooth interfacial charge transfer at the photoanode/electrolyte and electrolyte/counter electrode. Relative to the commonly used iodide/triiodide (I^−^/I_3_^−^) redox couple and platinum counter electrode in dye-sensitized solar cells (DSSCs), the sulphide/polysulphide redox couple and CuS counter electrode have been proposed to be suitable for metal chalcogenide (*e.g.*, CdSe)-based QDSSCs, respectively.^56,58,64^ The presence of Sn^2−^ (oxidized counterpart) in the sulphide redox couple causes the quick scavenging of the photogenerated hole in the CdSe QDs by the redox couple, and thus regenerating QDs. However, the greater concentration of polysulfide also contributes the back electron transfer process.^70^ Leakage of the liquid electrolyte unfortunately results in a medium loss for charge transfer. Thus, solid-state electrolytes might be considered, such as polysulfide integrated polyvinylpyrrolidone.^71^ On the other hand, the penetration of solid electrolyte in the entire photoanode still is a challenging issue to be tackled. ZnO has also been applied for the counter electrode, in which the metal sulfide (*e.g.*, CuS, PbS)-deposited ZnO nanorod counter electrode has shown better electrocatalytic activity than CuS (Table 2).^72,73^ The strategies to improve QDSSC performances by using ZnO nanostructured materials are summarized in Table 2.

**Table 2:** Strategies to improve QDSSC performances

Photoanode	Approach	QD	*I*_sc_ (mA cm^−2^)	*V*_oc_	Ff	*η* (%)	Ref.
Ultrasonic spray pyrolysis-synthesized ZnO	Good contact between ZnO/FTO or ZnO/QD	CdS	6.99	0.66	0.33	1.54	59
ZnO nanotaper	Tapering morphology on nanorod	Nitrogen-doped graphene	∼1.04	—	—	∼1.15	^62^
ZnO nanotube	Vertically aligned electrical pathways, light scattering	CdSe	2.09	0.44	0.41	0.44	^63^
ZnO hollow microspheres	Light scattering	Zn_*x*_Cd_1−*x*_Se	20.77	0.43	0.33	2.95	^66^
ZnO hollow microspheres/TiO_2_ passivation layer	Light scattering, reduced surface defects	CdSe/CdS	14.64	0.46	0.47	3.16	^67^
ZnO mesoporous nanoparticles	Reduced surface defects and enhanced light harvesting capability	CdS/CdSe/MnS passivation layer	13.74	0.6	0.44	3.7	^65^
ZnO nanorods	CuS/ZnO nanorods counter electrode	Cds/CdSe	14.48	0.76	0.38	4.18	^72^
TiO_2_ nanoparticles	PbS/ZnO nanorods counter electrode	CdS/CdSe/ZnS	13.28	0.633	0.566	4.76	^73^
ZnO nanowire	Minimizing the SeO_2_ layer on CdSe QDs	CdSe/CdS	16.0	0.72	0.41	4.8	^61^

In addition to pursuing high photoconversion efficiency, the design of the assembly process (including materials used for sealing) is a limiting factor for long-term stability in a real application, even though it may not directly affect the performance. Extensive evaluation under various conditions, such as thermal, light and humidity stresses, should also be carried out to determining its stability. Afterward, the appropriate architecture and engineering of QDs-sensitized solar cells should be done comprehensively to make its practical application more feasible.

#### 2.1.3 Perovskite-sensitized solar cells

Perovskite is a class of compound with the general formula ABX_3_, where A and B are cations with different sizes, and X is an anion. The first attempt that began the story of the perovskite-sensitized solar cells, or recently denoted as the perovskite-solar cell, (PSC)^74–77^ was made in 2009 by Kojima *et al.*, where two organolead halide perovskite nanocrystals (CH_3_NH_3_PbBr_3_ and CH_3_NH_3_PbI_3_) were found to efficiently sensitize TiO_2_ for visible-light conversion in photoelectrochemical cells.^78^ They reported that the solar energy conversion efficiency of CH_3_NH_3_PbI_3_, also known as methyl ammonium lead iodide (MAPI), is higher than CH_3_NH_3_PbBr_3_ with a value of 3.8% and 3.1%, respectively. After this breakthrough, many researchers focused on the development of MAPI-based PSCs. Their efficiency has significantly jumped from 3.8% to 20.7% in less than ten years.^79^

n-Type semiconductor oxides (such as TiO_2_, ZnO) are widely used as electron-transporting materials (ETMs) to extract and transport the photogenerated electrons. At the same time, they block the photogenerated hole to suppress charge recombination in perovskite bulk films.^80,81^ Considering their crucial role in the photovoltaic performance of PSCs, it is essential to control the characteristics of ZnO as the ETM layer, especially its morphology, interfacial properties, trap states, and energy level alignment.^82^

##### 2.1.3.1 Influence of the ZnO nanostructure

Variations in the nanostructures have a significant impact on three aspects of the perovskite films: (i) the perovskite layer morphology and loading, (ii) the quality of the ZnO/perovskite interface, and (iii) the quality of the perovskite itself.^81^ In other words, ZnO with different nanostructures will lead to other PSC performances by affecting the perovskite directly, which will define the resulting PCE of a PSC. Briefly, the one-dimensional nanostructured ZnO (*i.e.*, nanorods; NRs) showed better performances than ZnO nanoparticles (NPs). These results can be observed mainly due to the single crystal one-dimensional structures of ZnO provides direct electron pathways for the electronic transport in PSCs.^80^ On the other hand, electrons suffer many trapping-detrapping events in NPs structures, especially at the grain boundaries that slow down the electron transfer.^83–85^ However, the trend cannot be linearly determined as there are other factors affecting PCE, besides the interacting properties of the perovskite and ETM in PSCs.

##### 2.1.3.2 Influence of ZnO deposition and post-treatment method

Variations in the ZnO processing method may affect the interaction between the perovskite and ETM, which determines the overall PCE of the PSCs. Zheng *et al.*^86^ showed that ZnO NPs deposited using the spin coating method resulted in a low power conversion efficiency (PCE) due to the formation of a pinhole-surface that creates the defective interface, leading to a loss of carriers.^86^ The PCE of the PSCs could be increased by adding the post-treatment method on the prepared ZnO NPs. Duan *et al.* showed that the addition of the *in situ* thermal decomposition on the spin-coated ZnO was able to change the morphologies of ZnO from nanoparticles to interconnect the net-like structure, leading to a power conversion efficiency increase to 13.1%.^87^ A higher PCE could be acquired by using ZnO NR. Theoretically, NR can result in better PCE by providing a direct electron pathway with its one-dimensional structure. To improve the PCE even more, Mahmood *et al.*^88^ improved the conventionally low aspect-ratio (LAR) ZnO NR by directly introducing the PEI polymer as a capping agent during the hydrothermal growth process, which resulted in a high aspect-ratio (HAR) ZnO NR. This modification increases the PCE as NR with a large diameter, hindering the perovskite infiltrations in the ETM and resulting in an increase of PCE from 10.3% to 11.5%. An even higher PCE can be achieved by passivating the ZnO NR layer with Al_2_O_3_, along with the post-treatment of solvent-annealing in ethanol vapor. This particular method increases the carrier diffusion length, as well as the recombination resistance in PSCs, which can result in 17.3% efficiency.^89^

##### 2.1.3.3 Influence of dopants and doping for interface engineering

More improvements of a PSC device can be achieved by improving the ZnO electronic properties for ETM by doping. Doping is already known to be adequate in modifying the electronic properties of metal oxide semiconductors.^90^ The doping can also significantly affect the morphology of ZnO, and is usually used to increase the free charges and thus, conductivity in solar cells.^81^ In the case of ZnO, doping can be achieved by either replacing the Zn^2+^ cation or the O^2−^ anion. Cationic dopants are typically metals, whereas anionic dopants are non-metals. Replacing Zn^2+^ by a different cation is expected to affect the conduction band (CB) structure. The upper edge of the valence band (VB) consists of O^2−^ 2p bands and replacing O^2−^ with a different anion affects the VB energy. Therefore, the doping of ZnO can shift the Fermi level (EF) in the direction of the CB, which helps increase the conductivity and facilitate the work function.^81,91^

In the case of using ZnO, several studies have shown the success in doping ZnO for PSC application. Dopants, such as N,^88^ I,^92^ Al,^93^ Ga,^94^ and Mg,^95^ are the highly preferred n-type dopants for ZnO films. The work of Mahmood *et al.*^88^ achieved 16.1% PCE by altering the aspect-ratio of the ZnO NR, and also by doping ZnO with electron-rich nitrogen, which was proven to efficiently increase the conductivity of the oxide layer, reduce the internal resistance, and hence increase the electron density of the ETM. Zheng *et al.* reported iodine-doped ZnO as ETM.^92^ Through the treatment of introducing iodine, the usual hydrothermal process resulted in a wide-hexagonal-structure of ZnO:I nanopillars, making a compact and even planar ZnO:I thin film surface with few voids compared to ZnO NR arrays. Iodine-doping to ZnO also promotes an electron extraction from the perovskite layer by a more favourable work function of the ETM, leading to a PCE as high as 18.24% of the device. Al-doped ZnO (AZO) and Ga-doped ZnO (GZO) films are also excellent candidates for transparent conducting oxide materials because they are inexpensive, have suitable ionic radii, and show excellent optical transmission performance. The work of Mahmood *et al.*^93^ showed that Al doping could greatly enhance the carrier concentration and electron mobility of pure ZnO, which results in superior conductivity. In addition to that information, Dong *et al.*^96^ worked to improve ZnO NR by Al-doping, making AZO. The resulting Al-doped ZnO (AZO) is reported to have a higher conduction band, a higher electron mobility, and a higher electron density than ZnO. The study shows that the use of AZO resulted in an increase of the solar cell efficiency from 8.5% to 10.07%, making them the first to fabricate the highly efficient Al-doped ZnO nanorod-based PSCs (Table 3). Summary of several factors that affect to photovoltaic performance of MAPI-based PSCs is presented in Table 3.

**Table 3:** The effect of ZnO nanostructure, preparation method, doping type, and other factors on photovoltaic performance of MAPI-based PSCs

ZnO nanostructure	ZnO preparation and/or post treatment method	Doping element	PCE (%)	Ref.
Nanoparticles	Non-aqueous preparation		4.3	^97^
Nanoparticles	Spin-coating		7	^98^
Nanorods	Spin-coating		9.1	^99^
Nanorods	Spin-coating	Al	10	^96^
Nanorods	LAR nanorods		10.3	^88^
Nanorods	Hydrothermal self-assembly + interfacial defect passivation (atomic layer deposition of Al_2_O_3_ monolayers on the ZnO nanorods)		10.4	^100^
Nanoparticles	ZnO/ZnS core–shell structure, spin-coating		10.9	^86^
Nanoparticles	Spin-coating, followed by *in situ* thermal decomposition		13.1	^87^
Nanorods	HAR nanorods		11.5	^88^
Nanorods	LAR nanorods	N	11.6	
Nanorods	HAR nanorods	N	13.6	
Nanorods	Hydrothermal process	Mg	15.3	^95^
Nanorods	Introducing PEI as capping agent, HAR	N	16.1	^88^
Nanorods	ZnO NR Al_2_O_3_ passivation + solvent-annealing		17.3	^89^
Nanorods (nanopillars)	Hydrothermal process	I	18.2	^92^
Nanoparticle	Two-step radio-frequency magnetron sputtering	Ga	20.2*	^94^

A study by Chen *et al.*^94^ showed that doping ZnO NR with Ga increased the carrier concentration such that an even higher power conversion efficiency of 20.167% was obtained, with an exciting transmittance value of >87% in the range of 0.4–1.2 μm. Dong *et al.*^95^ also worked to make Mg-doped ZnO, which is supposed to restrain the charge recombination in PSCs, owing to the conduction band offset at the ZnO/perovskite absorber interface, which increases the device efficiency.

Besides improving the ZnO properties, doping can also improve the PSC performance by simultaneously enhancing the interface interaction between the perovskite and the ZnO, in other words: interface engineering. This is because the charge generation, separation, collection, and recombination mainly occur at the interfaces. Interface engineering would be necessary to lower the interfacial energy barriers for charge transport, to suppress charge recombination, and to improve the performance of the solar cell.^15^ For example, iodine-doping to ZnO resulted in a lower work function for efficient electron extraction from the perovskite into ZnO:I. This was achieved by reducing the photoluminescence decay life-time, which is favourable for inhibiting the charge recombination at the interface, leading to a remarkable enhancement of *J*_sc_ and FF, together with PCE.^92^ Iodine-doping to ZnO also enables a better electrical contact interface between the perovskite and ZnO:I for facile charge transport, which effectively prevents charge accumulation at the interfaces.^92^ The study made by Dong *et al.*,^101^ which improved ZnO to make AZO, was shown to improve the material as an ETM. It also indicates that the use of this compound could reduce the recombination at the ZnO NR/perovskite interface. Ga-doped ZnO was also shown to have a crater-textured surface structure that can increase the contact area between ETM and perovskite, which reduces the contact resistance and increases the transmission channel of the electrons.^94^ The Ga doping in ZnO could increase the carrier concentration that makes the electrons effectively fill the interface traps and decrease the interface trap density, which was beneficial for reducing the electron capture and preventing carrier recombination at the interface, and improving the electron transporting efficiency from the perovskite to ETLs. Doping Mg to ZnO was also shown to raise the conduction band offset (ΔEC) at the ZnO/perovskite interface, suppressing the charge recombination, leading to improvements in cell performance.^95^

### 2.2 Heterojunction solar cells

#### 2.2.1 Inorganic heterojunction solar cells

ZnO is widely being used in various forms of inorganic heterojunction solar cells, including quantum dot solar cells (QDSC), thin-film solar cells, and excitonic solar cells for multiple purposes (*e.g.*, buffer layer, or as the n-type semiconductor in the active layer). One of the earliest examples of ZnO utilization in a fully inorganic solar cell was in 1976 by Kazmerski *et al.*, utilizing the intrinsic ZnO layer and n-type ZnO:Al in a Cu(InGa)Se2:CDS thin-film solar cell.^102^ The intrinsic ZnO layer in this demonstration was used as a buffer layer between the active layer and cathode, and the ZnO:Al blend functioned as a front contact. As time progressed, people started to utilize ZnO as an n-type semiconductor component for the BHJ active layer, owing to its high electron mobility, as well as the wide band-gap of 3.37 eV at 300 K.^103^

One of the most commonly found fully inorganic heterojunction solar cells consists of a mix between ZnO and lead chalcogenides (PbX; *e.g*., PbS, PbSe, PbTe). PbX has emerged as an excellent material for photovoltaic devices, as it has a uniquely large dielectric constant and therefore large Bohr radii, thus resulting in a significant quantum confinement effect.^104^ The energy level of PbX also favors combination with ZnO, in which the LUMO of the PbX can be tuned to minimize the difference with the conduction band energy of ZnO. Leschkies *et al.* reported a heterojunction solar cell based on the planar heterojunction between the PbSe nanocrystals and ZnO thin film.^105^ Compared to the Schottky solar cell made with similar PbSe NCs, the heterojunction solar cell utilizing the ZnO thin film features larger photocurrents and *V*_oc_ value, with an overall power conversion efficiency (PCE) of 1.6%. They found out that the thermal annealing of ZnO (up to 450 °C) yields a positive effect on the electrical conductance and electron mobility of the ZnO film due to the reduced defects in the post-annealed ZnO. To increase the efficiency, they proposed the utilization of nanostructures instead of planar films, like the one they used in this demonstration. Nanostructures provide a larger interfacial area for exciton dissociation compared to thin films. Thus, an increase in the overall device's performances were expected. Later, ZnO in the form of nanoparticles (ZnO NPs) was used as a substitute for ZnO thin film in a similar set-up; this time with PbS quantum dots nanocrystals as the p-type material.^106^ Massive improvements can be seen in the PCE of the resulting cell, which almost doubles that of the planar ZnO cell (*η* = 2.94%).

Other than the lead-based p-type semiconductors, copper p-type semiconductors were often used in combination with n-type ZnO, including Cu_2_O,^107^ Cu_2_ZnSnS_4_ (CZTS),^108^ Cu(In,Ga)Se_2_ (CIGS),^109^ and several others. The copper-based solar cell shows high potential as a material for low cost and non-toxic solar cells, which is an advantage compared to the Pb or Cd based cells.^110^ In 2018, Zang *et al.* utilized a perfectly oriented, micrometer grain-sized Cu_2_O/ZnO thin film to fabricate a solar cell with a PCE of 3.17%.^110^ The combination of the two yields outstanding results as the energy level favours each other for excitonic solar cell application; *i.e.*, the conduction band minimum of Cu_2_O is slightly higher than ZnO, and the valence band maximum of ZnO is lower than Cu_2_O. However, up until now, the highest PCE from a combination of Cu_2_O and ZnO thin film is only around 3–4%, which is still far lower than the theoretical PCE of 20%.^110–112^ In order to improve the efficiency, the interaction between ZnO and Cu_2_O (or other p-type semiconductor component for that matter) has a paramount importance to be addressed. Although nanoparticles (NPs) provide a large interfacial area, their uneven distribution may present a lack of facile electron pathway, causing high electron recombinant losses. Thus, new strategies must be developed, one of which includes the modification of the nanostructure's morphology.

Currently, most of the research is put into developing vertically-aligned nanostructures, such as nanorods or nanowires, as they offer the greatest interfacial area, as well as a facile pathway for charge transport.^113^ A demonstration in 2017 by Perng *et al.* reported the achievement of high to short-circuit current density (*J*_sc_ = 9.53 mA cm^−2^) using chemical bath deposition of ZnO NRs, instead of the usual sputtering, in a ZnO NRs:Cu_2_O BHJ setup.^114^ The PCE was improved immensely when using the nanorods structure, reaching up to 0.861% compared to 0.107% of similar cells utilizing the ZnO thin film. ZnO nanowires (NWs):CdS was also introduced into the CIGS solar cell, with a consistent trend as the Cu_2_O solar cell (*i.e.*, improved performances in cell employing NWs compared to thin-film).^109^ This work also demonstrates the possibility of performance enhancement effect using the piezo-phototronic effect; that is, increased performance with a suitable external mechanical strain. In the PbS QDs solar cell, ZnO NWs is also utilized, resulting in a cell with photocurrents of over 20 mA cm^−2^, and efficiencies of up to 4.3%.^115^ Later, Wang *et al.* reported the optimization of ZnO NWs/PbS QDs solar cells by tuning the PbS QDs dimension to study the performance of the device in the short-wave infrared region.^116^ It turns out that the solar cell working in the short-wave infrared region exhibits high *V*_oc_, making it a potential candidate for future uses as bottom or middle sub-cells in multijunction solar cells.

Recently, three-dimensional nanostructures in the form of a core/shell structure have been massively exploited. A 3D core/shell structure with ZnO nanostructure as the core is another promising route for highly efficient solar cells due to its ability to allow decoupling of the electrical and optical properties, as well as enhanced light trapping in the solar cell structure.^117,118^ ZnO NWs and tin(ii) sulfide (SnS) were combined to create a core/shell structure in a flexible solar cell using PET as the substrate, yielding a PCE of 1.2%.^119^ The piezo-phototronic effect was again taken as a strategy to enhance the performances in this flexible device, with a conversion efficiency increase of 37.3% under a moderate vertical pressure of 320 KPa. Another demonstration of the core/shell nanostructure was demonstrated by Akram *et al.*, this time with the ZnO blocking layer and CZTS as the p-type semiconductor. The Al-doped ZnO/ZnSe core/shell nanorod arrays were grown from the ZnO seed layer, creating a solar cell with efficiency reaching 2.2%, which is a massive improvement compared to a similar cell using the planar ZnO and ZnS as a buffer layer (*η* = 0.16%).^120^ The core/shell structure with ZnO NWs/AgGaSe_2_ bulk heterojunction active layer was also fabricated.^121^ AgGaSe_2_, although not as relatively popular as other p-type semiconductors, offers a high absorption coefficient and convenient band-gap nature. It was discovered that the synthesis time has a direct effect on the core/shell structure diameter, *i.e.*, an increase in diameter was observed with a longer growing time. A flexible solar cell on top of the PET substrate was successfully made with a PCE of 1.74%. Careful optimization of the nanorod array's diameter, length and spacing must be done to increase the efficiency even further.^120,122^

The usage of the ZnO nanostructures in a fully inorganic solar cell is not limited to being a component in the active layer. ZnO nanorod arrays are used as an antireflection layer in CSZTSe and Si solar cells.^123,124^ A decrease in the average reflection from 7.76% to 2.97% was detected when switching from bare ZnO to ZnO NRs structure with 900 nm rod length in the CSZTSe solar cell. The trend shows better device performance as the nanorod synthesis time increases up to 9 h (*η* = 4.08%).^123^ It was hypothesized that the ZnO fill the voids and pores better as the synthesis time takes longer, resulting in more intimate interfacial conditions, thus creating a cell with better performance. The SEM image also shows that a longer synthesis time directly translates to longer nanorod length, which, in turn, is accompanied by decreased electrical resistance.

ZnO as a buffer layer was demonstrated in a Sb_2_Se_3_ solar cell, replacing CdS as the conventionally used buffer layer due to its toxic nature.^125^ The randomly oriented ZnO produced by spray pyrolysis induced a favourable crystal growth orientation of the Sb_2_Se_3_, resulting in a device with fewer interfacial defects and high efficiency of 5.93%. Aside from better performance, the fabricated cell also offers superior stability, with only minor performance degradation after 1100 h of damp-heat testing (for comparison, a similar cell utilizing CdS dropped its efficiency from 5.67% to 5.16% after just 100 h of testing). Very recently in 2019, ZnO NPs were employed in a PbS colloidal quantum-dots (CQDs) system, with additional treatment in the form of oxygen annealing to the ZnO NPs to passivate its defects.^126^ Oxygen annealing produced a cell with the highest performance, with an efficiency of 9.05% compared to 7.98% and 6.90% in ambient air and N_2_ atmosphere, respectively. This indicates that the introduction of O_2_ gas during annealing can reduce the surface defects originating from the oxygen vacancies in ZnO NPs.

Aside from the morphology adjustments, elemental doping and interfacial modification were also proven to be useful strategies for improving the device's performance. The localized surface plasmonic resonance (LSPR) is one of the strategies that is often implemented to increase the performance of a photovoltaic device. By modifying the size of a nanomaterial, it is possible to change and tune their band absorption.^127^ In 2015, plasmonic Ag nanocubes were introduced to the PbS:ZnO NW solar cell for further improvement in its performances, particularly in the infrared and visible light region because of the plasmonic enhancement of light absorption in the range of 700–1200 nm.^128^ As a result, the PCE improved from 4.45% to 6.03% after the addition of Ag nanocubes at 25% coverage. An excess in the addition of Ag nanocubes, however, results in performance decline. This is due to the suppressed charge separation because of the hole–electron recombination at the surface of the nanocubes, and the possibility of Ag nanocubes aggregation. In its use as an electron extraction layer, the caesium-doped ZnO nanoparticles were synthesized and used in the PbS colloidal QDs system.^129^ Elemental doping in the form of caesium doping increases the cell efficiency by up to 10.43% with 5% doping of Cs, compared to 9.20% efficiency in the cell with pristine ZnO as the electron transport layer. The addition of Mg doping has been implemented to create a Zn_0.9_Mg_0.1_O layer using the sol–gel method. As an interlayer between Sb_2_Se_3_ and ZnO, it was found to increase the PCE from 3.22% to 4.45%, which was attributed to the interlayer's ability to passivate defects and reduce recombination losses.^130^ Cobalt doping was used in a band-alignment approach to optimize the performance of the CuO nanostructure and ZnO NRs solar cell, using a low-temperature chemical bath deposition technique (Table 4).^131^ The device performances of a fully inorganic solar cell using ZnO are summarized in Table 4.

**Table 4:** Device performances of a fully inorganic solar cell using ZnO

Solar cell architecture	ZnO role	*V*_oc_ (V)	*J*_sc_ (mA cm^−2^)	FF (%)	PCE (%)	Additional note	Ref.
ITO/ZnO film/PbSe NCs/Au	Active layer n-type component	0.45	15.7	27	1.6		^105^
ITOZnO NPs/PbS QDs/Au	Active layer n-type component	0.59	8.9	56	2.94		^106^
ITO/ZnO film/Cu_2_O/Ag	Active layer n-type component	0.56	11.4	49.8	3.17	Perfectly oriented micrometer grain-sized Cu_2_O was used	^110^
AZO/ZnO film/Cu_2_O/Au	Active layer n-type component	0.71	9.69	60	4.13		^112^
ITO/Cu_2_O/ZnO NRs	Active layer n-type component	0.15	7.03	33	0.33		^132^
ITO/Cu_2_O/ZnO NRs/ITO	Active layer n-type component	0.34	7.77	39.5	1.05	Ag mirror was used at the backside as a photon reflector	^114^
ITO/ZnO NWs/CdS/CIGS/Mo	Active layer n-type component	0.61	26.44	71.16	11.4	Rigid device with glass as a substrate. Vertical pressure was applied to induce the piezo-phototronic enhancement effect	^109^
FTO/ZnO/ZnO NWs/PbS QDs/Au	Active layer n-type component	0.464	28.5	52.8	6.98	Optimized PbS QDs dimension	^116^
ITO/ZnO NWs/SnS/Ag/EVA	Active layer n-type component	0.75	4.69	48	1.65	Core/shell structure between ZnO NWs and SnS. The flexible device using PET as a substrate. Vertical pressure was applied to induce the piezo-phototronic enhancement effect	^119^
FTO/ZnO/Al:ZnO NRs/ZnSe/CSTZ/Cu_2_S	Active layer n-type component	0.49	10.46	43	2.2	Core/shell structure between Al-doped ZnO NRs and ZnSe	^120^
ITO/ZnO NWs/AgGaSe_2_/Cu	Active layer n-type component	0.098	29.4	60.25	1.74	Core/shell structure between ZnO NWs and AgGaSe_2_	^121^
FTO/ZnO NWs:Ag nanocubes/PbS QDs/Au	Active layer n-type component	—	—	—	6.03	At 25% Ag nanocubes coverage	^128^
FTO/ZnO NRs:Co/CuO/MoO_3_/Au	Active layer n-type component	0.47	9.49	48.4	2.11		^131^
ZnO NRs/AZO/ZnO/CdS/CZTSe/Mo	Antireflection layer	0.35	22.22	53	4.08		^123^
FTO/ZnO/Sb_2_Se_3_/Au	Buffer layer	0.39	26.2	57.8	5.93	9 h of growing time	^125^
FTO/ZnO/Zn_0.9_Mg_0.1_O/Sb_2_Se_3_/Au	Buffer layer	0.36	26.2	48	4.45		^130^
ITO/sputtered ZnO/PbS QDs/Au	Electron transport layer	0.47	19.45	42.4	3.87	Fully flexible device grown on top of PET substrate	^133^
ITO/ZnO NPs/TBAI-PbS/EDT-PbS/Au	Electron transport layer	0.55	24.2	63.8	8.55		^134^
ITO/ZnO NPs/TBAI-PbS/EDT-PbS/Au	Electron transport layer	0.59	25.0	61.36	9.05	ZnO NPs underwent oxygen annealing instead of regular annealing	^126^
ITO/Cs-ZnO NPs/TBAI-PbS/EDT-PbS/Au	Electron transport layer	0.59	26.2	67.5	10.43	5% of caesium doping	^129^

With 10% cobalt doping, a PCE of 2.11% was obtained due to the lowered band-gap of the ZnO layer.

Interfacial modification usually involves electrodeposition of another material to improve the interfacial condition. One of the most frequently used substrates for this purpose is carbon-based nanostructures, with graphene quantum dots gaining massive popularity due to its excellent luminescent property, good solubility, and pronounced quantum confinement effect.^135^ Very recently, an example of interfacial modification was demonstrated using electrodeposited graphene-oxide onto a ZnO film, followed by thermal annealing to improve the relatively modest performance of the ZnO/Cu_2_O heterojunction solar cells.^107^ Introduction of the graphene oxide nanosheets results in higher photo-electrical properties due to their strong interface properties. A decrease in the ZnO band-gap was also observed by the addition of reduced graphene oxide, which resulted in improved solar cell performance.^136^ Chuang *et al.* managed to enhance the performance of ZnO NPs/PbS QDs cell through band alignment by utilizing various ligand treatments.^134^ They discovered that, by using tetrabutylammonium iodide (TBAI) and 1,2-ethanedithiol (EDT) as ligands for solid-state ligand exchange, a shift in the first exciton absorption peak to higher value was detected. Higher stability, *V*_oc_, and *J*_sc_ were observed after ligand addition. After layer stacking optimization between TBAI-PbS and EDT-PbS, a ∼35% increase in PCE was detected compared to the cell using only TBAI-PbS.

#### 2.2.2 Hybrid organic/inorganic heterojunction solar cells

##### 2.2.2.1 ZnO as active layer in hybrid solar cells

Organic–inorganic hybrid solar cells (HSCs) have been receiving significant attention due to its mechanical flexibility and potential to be made at a low cost.^137^ These hybrid solar cells combine two components to convert sunlight into electrical charge: (i) a conjugated polymer as an organic semiconductor, serving as a light harvester and electron donor, and (ii) an inorganic semiconductor acting as the electron acceptor.^138^ ZnO has been widely used to replace the electron acceptor organic semiconductor present in fully organic solar cells (OSCs), mainly due to the observed higher electron mobility in the inorganic component of HSCs compared to most of the currently available n-type organic semiconductors, as well as higher physical and chemical stability.^52^ The overall energy production mechanism is very similar to that of OSCs, which involves exciton generation due to light illumination, which will then diffuse to the donor/acceptor interface within a certain diffusion length. The difference between the HOMO (highest occupied molecular orbital) and LUMO (lowest unoccupied molecular orbital) between the donor and acceptor material provides a driving force to overcome the binding energy of excitons generated in the previous step, dissociating them onto two separate free carriers by charge transfer. The positive and negative charges will then go to the cathode and anode, using continuous charge pathways provided by the acceptor and donor materials, respectively.^139^ Many nanostructures of ZnO have been investigated for HSCs applications, including nanoparticles, nanowires and nanorods.^140–143^ In this review, we will focus on discussing the three main device architectures of organic–inorganic HSCs, which include: (i) bulk heterojunction HSCs with randomly dispersed nanocrystals, (ii) HSCs with vertically aligned nanostructures, and (iii) HSCs with organic–inorganic bilayer structure.^144^

The utilization of ZnO in bulk heterojunction solar cells was first reported in 2004. Beek *et al.* managed to utilize separately prepared nanocrystalline ZnO (nc-ZnO) with a diameter of ∼5 nm *via* hydrolysis and condensation of zinc acetate dihydrate by KOH in methanol to create a bulk heterojunction HSCs, along with poly[2-methoxy-5-(3′,7′-dimethyloctyloxy)-1,4-phenylenevinylene] (MDMO-PPV) as the organic p-type semiconductor. This configuration managed to reach a power conversion efficiency (PCE) *η* = 1.6% at 0.71 sun equivalent intensity, and remained relatively stable at higher intensity of 1.7 sun equivalent (*η* = 1.4%).^140^ The forward current density observed in ZnO:MDMO-PPV was significantly higher than the one in pristine MDMO-PPV in a similar configuration, indicating that the presence of nc-ZnO does indeed provide a continuous pathway for electron transport.^140^ The same group later tried to substitute MDMO-PPV to poly(3-hexylthiophene) (P3HT), due to it possessing higher hole mobility.^145^ However, the HSCs with P3HT shows a lower PCE of 0.9% when compared to the nc-ZnO:MDMO-PPV cell the group made earlier.^140,145^ Previously, it was proposed that the main limiting factor of the organic–inorganic HSCs lies in the hole mobility of the organic phase. Thus, the lower result came as a surprise.^146^ It was later found that the presence of the hydrophilic pre-synthesized ZnO inside the polymer blend may negatively influence its ability to crystallize, thus reducing the hole mobility inside the polymer. An investigation using AFM shows that ZnO particles are not perfectly evenly distributed, especially at the interface with PEDOT/PSS, creating a thin layer consisting of pure polymer. This layer will hinder the excitons generated within the PEDOT/PSS to reach the ZnO interface to create a charge, reducing its internal quantum efficiency (IQE).^147^ From these studies, it can be concluded that the lack of homogeneity in the particle dispersion caused by the nc-ZnO-based HSCs manufacturing process itself, as well as the lack of an intimate mixture between the organic and inorganic phase at the interface, contributes to an inefficient electron transport, reducing the device's overall efficiency.^144,148^

An alternative route of the ZnO HSCs fabrication can be done by utilizing ZnO in its precursor form, usually in the form of diethylzinc.^149^ The precursor is first solved onto an organic solvent, and then cast into a thin film together with the polymer. When the mixture reacts with moisture in the air, the well-dispersed ZnO particles are formed across the polymer film *via* hydrolysis reaction.^150^ Thermal annealing near the polymer's glass transition temperature is usually employed afterwards to improve the polymer crystallinity, which will improve the hole mobility and packing.^145^ This method was first demonstrated using TiO_2_ as the inorganic n-type semiconductor, but shows poor results due to the high temperature required to form crystalline TiO_2_.^149^ ZnO, on the other hand, crystallized at a much lower temperature (∼110 °C). Using this precursor-based method, as well as the employment of the post-fabrication thermal annealing, more evenly distributed ZnO nanoparticles across the film were observed. This enabled the previously modest performance nc-ZnO:P3HT cell to be improved immensely, increasing its PCE by 0.5% (*η* = 1.4%).^151^ The three-dimensional morphology of ZnO was later employed with P3HT using the precursor method, yielding *η* of 2% in the cell with 167 nm active layer thickness. This study highlights the effect of thickness on the cell's performance, in which thinner cells tend to exhibit lower properties. The relatively poor performance of the thin ZnO:P3HT HSCs was caused by the inefficient charge generation and charge transfer due to the coarse phase separation, thus presenting a lack of continuous pathway.^147^

The usage of the ZnO nanocrystals was also demonstrated as an electron transport layer in a P3HT:PbS BHJ hybrid solar cell, with the CdSe quantum dot acting as a buffer layer.^152^ Here, the ZnO nanocrystals act as the electron transporting medium between the active layer and the ITO cathode, with the CdSe quantum dot layer bridging the energy difference between ZnO and the active layer due to its energy level lying in-between ZnO and P3HT:PbS. ZnO nanocrystals were synthesized separately and annealed at 230 °C for 20 minutes to promote crystallization. By tuning the size of the CdSe quantum dots, the best device of this approach managed to reach an efficiency of 2.4%. The earlier demonstration had utilized the ZnO nanowire for the same purpose, displaying a fourfold photovoltaic performances compared to the device without a ZnO nanowire.^142^

Besides the randomly dispersed nanoparticles, more ‘controlled’ and aligned nanostructures (such as nanorods and nanowires) are widely being developed.^153^ The vertically aligned nanostructures have been regarded as one promising candidate because the vertical alignment provides a higher interfacial area between the organic and inorganic material, creating a highly efficient pathway for the electron transport.^154^ One advantage of ZnO compared to other often utilized inorganic materials is the fact that the vertically aligned ZnO can be grown easily in many substrates *via* low-cost techniques, such as hydrothermal and solution methods.^155–157^ Olson *et al.* first demonstrated the idea of utilizing vertically aligned ZnO nanofibers in combination with P3HT to create an HSC device, in which the resulting device exhibited *η* of 0.53%.^158^ The modest results are believed to be caused by several unoptimized parameters, such as the spacing between ZnO nanofibers (100 nm), which is too large when compared to the typical exciton diffusion length in P3HT (10–20 nm), resulting in an inefficient charge separation.^148^ The same group later substituted the solvent used in P3HT solution from chloroform to dichlorobenzene. The device with dichlorobenzene shows a better result due to an improved polymer infiltration and polymer chain ordering.^159^

Much like the nanoparticle structures, annealing near the polymer's melting temperature for a short time provides more time for the polymer to arrange between the nanofibers. This provides a better, more intimate interface between the two phases, therefore reducing the geminate recombination.^160^ An increase in the polymer crystallinity was also observed. By such optimization, as well as the optimization of the ZnO backing layer thickness, Baeten *et al.* managed to report a noticeable increase in the device performance of the ZnO nanorod arrays coupled with P3HT.^160^ It is important to note that the best device produced by thermal annealing only underwent this process for 1 min at 225 °C, as further annealing (up to 15 min) showed diminishing performance due to unfavourable polymer chain ordering.^160^ An optimized performance of PCE 1.44% was later demonstrated in an inverted cell structure with 0.08 mol L^−1^ concentration of the precursor, 5 h of the hydrothermal time, followed by 100 °C thermal annealing, and spin-coating 3 layers of PEDOT:PSS.^156^ Surface engineering of the ZnO nanostructures may also be employed to further increase its performance (Table 5). Device performances of organic-inorganic hybrid solar cells using ZnO as active layer component are summarized in Table 5.

**Table 5:** Device performances of organic–inorganic hybrid solar cells using ZnO as active layer component

ZnO structure	Organic semiconductor	*V*_oc_ (V)	*J*_sc_ (mA cm^−2^)	FF (%)	PCE (%)	Additional note	Ref.
Nanoparticles	MDMO-PPV	0.814	2.4	59	1.6		^140^
Nanoparticles	P3HT	0.8	2.2	46	0.9		^145^
Nanoparticles	P3HT	0.83	3.5	50	1.4	Made using precursor method instead of pre-synthesized nanocrystals	^151^
3D network	P3HT	0.75	5.2	52	2.0		^147^
Nanofibers	P3HT	0.44	2.2	56	0.53		^158^
Nanorods	P3HT	0.543	2.67	53	0.76	Thermal annealing at 225 °C for 1 min	^160^
Nanorods	P3HT	0.312	10.5	45	1.44	0.08 mol L^−1^ concentration of precursor, five h of hydrothermal time, 100 °C thermal annealing, and spin-coating three layers of PEDOT:PSS. Inverted cell	^156^
Nanowires	P3HT	0.37	2.71	54	0.57	Sn doping of 1% mol ZnO. SQ2 dye used as the interlayer	^161^
Surface modified nanorods	P3HT	0.72	1.94	53	0.93	(*E*)-2-cyano-3-(5′-(4-(Dibutylamino)styryl)-2,2′-bithiophen-5-yl)acrylic acid grafted as p-type ligand on the ZnO NRs surface	^162^
Surface modified nanorods	P3HT	0.47	1.43	48.7	0.32	0.1 M KOH used as etching agent	^163^
Trilaminar nanorods	P3HT	0.44	5.57	54	1.32	ZnO/ZnS/Sb_2_Se_3_ trilaminar structure was used	^164^
Monolayer	P3HT	0.371	0.52	49	0.07	150 °C heating for 10 min under N_2_ atmosphere	^165^
Chemically modified monolayer	P3HT	0.7	1.27	55.6	0.49	Zn_0.75_Mg_0.25_O composition yields the best result	^166^
Nanowires	CuPc:C60	0.46	3.86	30	0.53	ZnO utilized as electron transport layer	^142^
Nanoparticles	P3HT:PbS	0.61	7.75	50.2	2.4	ZnO utilized as electron transport layer. CdSe QD used as buffer layer. Inverted cell	^152^

In 2017, Dkhil *et al.* incorporated a p-type semiconductor ligand ((*E*)-2-cyano-3-(5′-(4-(dibutylamino)styryl)-2,2′-bithiophen-5-yl)acrylic acid), grafted as the interfacial surfactant on ZnO nanorods, to improve the interfacial bonding between the two phases in the ZnO-NR:P3HT cell.^162^ The best device produced by this approach yields a PCE of 0.93%, which is better than the ZnO-NR:P3HT cell without any surfactant modification. To further improve the performance, the development of a suitable n-type semiconductor ligand was proposed by the same group to enhance the compatibility between the organic–inorganic phases.^162^ It should be noted that the ligand-related approaches can also be applied with other ZnO/polymer HSCs architectures, and has been demonstrated in both dispersed nanoparticles and vertically aligned nanostructures alike.^162,167^ Chemical etching with protonic and anionic agents were also demonstrated, and showed positive results owing to the favourable ZnO nanorod morphology. The KOH-treated ZnO nanorods showed the most significant improvement due to the defect quenching phenomenon provided by KOH.^163^ The unique surface structure in the form of the trilaminar ZnO/ZnS/Sb_2_Se_3_ nanotube (NTs) arrays was used in conjunction with P3HT, where ZnO was used as a buffer layer and Sb_2_Se_3_ as sensitizer.^164^ The structure was reported to be able to suppress carrier recombination and increase electron collection efficiency due to better energy level alignment of the trilaminar structure with P3HT, thus giving a rise in PCE of 1.32%.

The bilayer structure is another important and widely studied architecture of organic–inorganic HSCs. This architecture first deposits a ZnO layer above the electrode surface, followed by the deposition of the p-type organic semiconductor, and topped by the top electrode. Unsurprisingly, this simple architecture usually yields lower performance than two previously discussed architecture (*e.g.*, dispersed nanoparticles and vertically aligned nanostructures), mainly due to the smaller interface area available for electron transport.^168^ Regardless, various efforts have been made to improve the properties of such a simple structure, which includes doping and surface modification.^165,168,169^ Olson *et al.* first reported the effect of interfacial modification on polymer/ZnO bilayer devices in 2008. The demonstration shows that the ZnO/P3HT device, which undergoes heating at 150 °C exhibits much higher *V*_oc_ (difference of around 200 mV) compared to the device treated with ozone/UV, owing to changes in the interfacial dipoles, causing a band alignment shift at the interface.^165^ The efficiency rises from 0.02% to 0.07% when switching from ozone/UV treatment to heating, although their performance is still lower than similarly treated ZnO nanorods-based devices.^158,170^ Chemical doping of ZnO with other elements, such as Mg, has also been successfully demonstrated and yields favorable results.^166^

##### 2.2.2.2 ZnO as the cathode buffer layer in organic solar cell

In addition to being an electron acceptor in HSCs, ZnO is also often used in fully organic solar cells (OSCs) as the cathode buffer layer (CBL). Similar to HSCs, the conjugated polymer takes the role of being the p-type semiconductor. However, instead of utilizing the inorganic component, fullerene-derivatives (*e.g.*, PCBM) are used as the electron acceptor and electron transporting material.^171^ It has been known that OSCs, especially the conventional type, exhibit low stability due to the susceptibility of its metal cathode (*e.g.*, Al) used in OSCs to moisture and air. The insertion of ZnO materials into the interface between the active layer and cathode as a buffer layer has been demonstrated in many cases. The approach shows an overall positive result in improving the stability of the cell. On the other hand, the favourable energy band of ZnO also makes it very suitable for this purpose, as the lowest conduction band energy of ZnO is lower than the LUMO of typical fullerene-derivative semiconductors. The highest valence band energy of ZnO is lower than the HOMO of polymer donor semiconductors, such as P3HT.^172,173^ This essentially means that ZnO can both help to extract and collect electrons in the fullerene acceptor, while also blocking the unwanted reverse flow of the hole from the polymer donor into the cathode, preventing the generation of a leakage current. Usage of ZnO was demonstrated in both conventional and inverted solar cell structures, and the two will be discussed in this review.

In 2011, Jouane *et al.* demonstrated the introduction of a ZnO layer onto the active layer of a P3HT:PCBM conventional organic solar cell *via* rf magnetic sputtering. The deposition of the ZnO layer causes no functional damage to the photoactive layer, and increases the cell efficiency from 2.16% to 2.34%.^174^ Interface engineering between the backing layer and metal cathode is also needed to improve the device performance in both OSCs and HSCs. The self-assembled monolayer (SAM) has been implemented in photovoltaic device fabrication to tune the characteristics of the ZnO surface.^175^ Modification of the ZnO surface in the CBL/cathode interface with the carboxylic acid-based SAM has been demonstrated using various different metals as the cathode.^176–178^ They found out that the dipole direction and chemical bonding between the CBL/cathode are two key factors in improving the device performance, in which the favorable dipole generated ohmic contacts and will improve the device efficiency.

Where ZnO-based CBL truly found its uses, however, is in the inverted structure of the OSCs. The inverted structure eliminates the mandatory need of the PEDOT:PSS layer, which may cause etching on the ITO glass, resulting in a degradation of the cell.^179^ The inverted structure also enables a low-work-function metal (like Al) to be replaced by an air-stable high-work-function metal, such as Ag or Au, resulting in much greater stability. Earlier fabrications of inverted OSCs generally reported lower PCE than conventional cells, but recent studies have shown that it is possible to fabricate high performance and high stability inverted cells.^180^ However, when using P3HT:PCBM based OSCs, the reported PCEs are mostly less than 5%.

To combat this, a new fullerene derivative with higher LUMO is being developed to increase the overall *V*_oc_ and therefore device efficiency. An example of the recently developed fullerene derivative is the indene-C_60_ bisadduct (ICBA), which has a LUMO of −3.74 eV compared to −3.91 eV of PCBM.^181^ A blend of ICBA and P3HT has been implemented in conventional solar cells and yielded an impressive PCE of 5.44%, which is superior to that of the P3HT:PCBM device with PCE of 3.88%.^182^ When used in the inverted solar cell, a *V*_oc_ of 0.82 V was achieved, which is higher than the *V*_oc_ limitation of the P3HT:PCBM blend of 0.65 V.^181^ Fabrication of a similar inverted cell using inkjet printing instead of the spin coating was demonstrated by Ganesan *et al.* in 2019. However, the fabricated cell performance still falls short (*η* = 4.7%) due to the unoptimized printing parameters.^183^ Using the same logical approach, the [6′6]-phenyl C_70_-butyric acid methyl ester (PC_70_BM) was blended with poly[5,5′(4,4′-bis-(2-ethylhexyl)-dithieno[3,2-*b*:2′,3′-*d*]germole)-*alt*-1,3(5-octyl-4*H*-thieno[3,4-*c*]pyrrole-4,6(5*H*)-dione)] (P-Ge) to form an active BHJ layer in the inverted solar cell, in which the resulting solar cell exhibited a PCE of 7.3%, without compromising its stability.^184^

Various nanostructure parameters of ZnO, such as the size and surface area, also play an integral part in determining the overall device performance.^185^ There have been many demonstrations of low-dimensional ZnO nanostructures utilized in OSCs, both in the form of simple nanoparticles and one-dimensional nanostructures. Hau *et al.* reported the utilization of ZnO nanoparticles in the inverted OSCs structure, with the P3HT:PCBM active layer and PEDOT:PSS/Ag as an anode. The resulting device with the nanoparticle arrangement of ZnO exhibited higher efficiency compared to ZnO deposited using the sol–gel method in a similar cell setup.^176^ It should also be noted that the ZnO NP is synthesized at room temperature with a solution method, compared to the bulk ZnO processed with high temperature sol–gel method. However, a similar problem with the NP-based HSCs arises, in which the randomly dispersed nanoparticles do not provide an adequate pathway for charge transport, which may limit the device efficiency due to a higher rate of electron recombination losses compared to aligned, one-dimensional nanostructures.^113^ Recently, annealing-free ZnO nanoparticles were fabricated by Jung *et al.* in 2018.^155^ This demonstration not only yields a respectable PCE of 7.41%, but more importantly, also demonstrated the possibility of skipping the annealing process, which may damage the substrate and active component during the fabrication.

One-dimensional nanostructures have been regarded as a promising approach to fabricate efficient OSCs. Such 1D structure can provide a direct and ordered pathway from the photogeneration site into the metal cathode, reducing the electron recombination losses, thus increasing the overall device performance.^113^ Takanezawa *et al.* demonstrated the usage of the ZnO nanorods array with controlled dimension, and coupling the nanorods with the P3HT/PCBM polymer blend to produce OSCs with PCE of up to 2.7%.^186^ This experiment uses the spin-coating method to reliably control the organic layer thickness, as well as the post-fabrication thermal annealing process. Nanorods in this experiment were made *via* the hydrothermal method, yielding a nanorods array with a diameter of 20–40 nm and length of ∼0.3 μm. From this work, it was concluded that the nanorod's length and the organic layer's thickness play an important role in determining the device's efficiency, in which performances were found to improve with the increase of the average NR length.^186^ Chou *et al.* reported in 2009 that the slow-drying process, which lengthens the polymer's solidification time, can improve the FF and PCE of the inverted ZnO:P3HT/PCBM (*η* = 3.58%) due to the improved polymer crystallinity and infiltration of the photo-active layer.^187^

Nevertheless, compared to 1D structures, the nanoparticle structure provides better electron collection from the photoactive layer compared to the 1D nanostructure. Thus, the combination of the two, forming a bilayer of ZnO NR-ZnO sol gel was demonstrated in 2015 by Ambade *et al.*, in which the fabricated P3HT:PCBM cell exhibited a PCE of 3.70%.^188^ This novelty was explored to demonstrate the possibility of such efficient bifunctional CBL, in which the sol–gel layer first efficiently collects the electron, and is then transported effectively by the nanorods into the cathode. Another novel 1D nanostructure of ZnO that exhibited similar nanostructure to that of the previously mentioned bilayer CBL was also explored. Sekine *et al.* synthesized ZnO nanoridges in a thin film with a peak height of ∼120 nm and distance between ‘valleys’ of around 500 nm. The synthesis of the ZnO nanoridges was done using a sol gel method similar to that of the planar ZnO synthesis, but different annealing conditions.^189^ The presence of the ZnO nanoridges structure shows a significant performance improvement compared to that of the planar ZnO, with the P3HT:PCBM ZnO nanoridge inverted solar cell reaching an efficiency of 4%. More recently in 2017, Ryu *et al.* demonstrated the utilization of ZnO nanoridges with a low static annealing temperature of 150 °C, which makes the temperature low enough for co-processing plastic substrates for flexible device applications.^190^ A PCE of 6.24% was obtained using said ZnO combined with a PTB7-F20:PCBM active layer.

Aside from altering the nanomorphology of the CBL, better performance can be achieved by modification of the ZnO itself, which may include surface modification or elemental doping. Surface modification ranges from the introduction of dyes,^191^ SAMs (self-assembled monolayers),^192^ and fullerene-derivatives.^193^ The addition of C_60_-SAMs has been reported to enhance the device performance by up to 20% increase, owing to the enhanced interfacial exciton dissociation energy.^194^ Another fullerene-derived organic molecule, crosslinked-[6,6]-phenyl-C_61_-butyric styryl dendron ester (C-PCBSD) was used to modify the surface of ZnO, yielding a PCE of 4.4% when incorporated into the P3HT:PCBM OSC,^195^ and 6.22% in the P3HT:ICBA OSC,^181^ as well as a significantly improved lifetime in both compared to cells without surface modification.^182^ Surface modification with graphene oxide and reduced graphene oxide has also been demonstrated, yielding an inverted cell with PCE as high as 9.49% in the cell utilizing *in situ* thermal reduced graphene oxide.^196,197^ Silane has also been used as a capping agent for ZnO NPs to prevent aggregation. This is an attempt to improve the solar cell's stability, and the quality of the interfacial contact between the active and the buffer layers. Wei *et al.* demonstrated the synthesis of the 3-aminorpropyltriethoxysilane-capped ZnO nanoparticles, which can remain stable in air for more than one year.^198^ Recently, the down-shifting effect has been proven to be able to increase the solar cell performance by converting UV light to visible light.^199^ Lanthanide down-conversion material, with the ability to absorb UV light and re-emit it in the visible region, was added to the ZnO electron transport layer, so that the re-emitted light matches the absorption energy level of the active layer material.^200^ Here, Eu(TTA)_3_phen (ETP) was used as the down-conversion material, with PTB7-Th:PC_71_BM as the active layer, resulting in a cell with PCE of 9.22% and 70% higher stability compared to the cell with pristine ZnO. In 2020, Shen *et al.* demonstrated the fabrication of ITO-less solar cells, using oxygen-doped Ag and plasmonic Ag@SiO_2_ as a countermeasure for the lack of the ITO layer, taking advantage of both micro-resonant cavity and plasmonic effect.^201^ The optimized cell displays a PCE of 8.04%, which is 36.27% higher than the ITO-based cell.

Elemental doping with elements, such as La, In and Ga, has been reported to dramatically increase the efficiency of organic solar cells, with Ga-doped ZnO (GZO) reportedly increasing the cell PCE by 110%, owing to the higher electron conductivity and better wettability due to the favourable surface morphology.^202–204^ More recently in 2016, Li-doped ZnO has been utilized as CBL in the P3HT:PCBM cell, and managed to obtain 30% improvement from the non-doped ZnO CBL layer due to the enhanced electron mobility, smoother surface morphology and better energy band matching in the Li–ZnO CBL.^205^ This approach has also been demonstrated in the non-P3HT:PCBM cell. For example, in 2018, Hf–In–ZnO was used as an electron transport layer in the inverted PTB7:PC_70_BM solar cell, yielding a solar cell with an efficiency of up to 4.15%, with twice the lifetime of a similar OSC with PFN as a buffer layer, because the Hf atoms have a strong thermodynamic tendency to form metal oxides, suppressing the dissociation of Hf–In–ZnO (Table 6).^206^ Summary of device performances of organic solar cells using ZnO as cathode buffer layer is shown in Table 6.

**Table 6:** Device performances of organic solar cells using ZnO as a cathode buffer layer

Cathode	Anode	Active layer	*V*_oc_ (V)	*J*_sc_ (mA cm^−2^)	FF (%)	PCE (%)	Additional note	Ref.
ZnO/Al	ITO/PEDOT:PSS	P3HT:PCBM	0.63	7.99	45.7	2.34		^174^
ZnO/SAM/Al	ITO/PEDOT:PSS	P3HT:PCBM	0.65	11.10	63.0	4.60	Mercaptoundecanoic acid (MUA) used as SAM	^178^
ITO/ZnO	Ag	P3HT:PCBM	0.56	11.22	47.5	2.97		^207^
ITO/ZnO	MoO_3_/Au	PSiF-DBT:PCBM	0.90	5.03	60	3.80		^208^
ITO/ZnO	PEDOT:PSS/Ag	P3HT:ICBA	0.82	10.6	55	4.81		^181^
ITO/ZnO	MoO_3_/Ag	P3HT:ICBA	0.83	9.57	60	4.7	Inkjet-printing method was used	^183^
ITO/ZnO	MoO_3_/Ag	P-Ge:PC70BM	0.85	12.6	68	7.3		^184^
ITO/ZnO NPs	PEDOT:PSS/Ag	P3HT:PCBM	0.62	10.69	54.2	3.61		^176^
Graphene/ZnO NPs	MoO_3_/Ag	PTB7-Th:PC71BM	0.76	15.63	63	7.41	PET was used as substrate, making this cell a fully flexible device. Annealing-free process was used	^155^
ITO/ZnO NFs	Ag	P3HT:PCBM	0.48	10	43	2.03		^158^
ITO/ZnO NRs	Ag	P3HT:PCBM	0.57	9.6	50	2.70	Optimized nanorods dimension	^186^
ITO/ZnO NRs	Ag	P3HT:PCBM	0.53	11.7	58	3.58	Slow-drying process was employed	^187^
ITO/ZnO NRs-ZnO SG	MoO3/Ag	P3HT:PCBM	0.61	10.66	57	3.70		^188^
ITO/ZnO nanoridges	V2O5/Al	P3HT:PCBM	0.60	10.76	62	4.00		^189^
ITO/ZnO nanoridges	PEDOT:PSS/Ag	PTB7-F20:PC71BM	0.69	15.67	57	6.24	Low temperature static annealing (150 °C) was employed	^190^
ITO/ZnO	PEDOT:PSS/Ag	P3HT:PCBM	0.60	12.8	58	4.4	C-PCBSD utilized as SAMs	^195^
ITO/ZnO	PEDOT:PSS/Ag	P3HT:ICBA	0.84	12.4	60	6.22	C-PCBSD utilized as SAMs	^181^
ITO/rGO/ZnO NPs	MoO_3_/Ag	P3HT:PCBM	0.63	9.49	63.4	3.77		^197^
ITO/GO/rGO/ZnO NPs:rGO	MoO_3_/Ag	PTB7-Th:PC71BM	0.78	18.61	65.4	9.49	*In situ* thermal reduction and annealing was used to synthesize reduced graphene oxide	^196^
ITO/ZnO NPs:APTMS	MoO_3_/Al	PTB7-Th:PC71BM	0.80	16.67	68	9.07		^198^
ITO/ZnO:ETP	MoO_3_/Ag	PTB7-Th:PC71BM	0.76	17.48	67.6	9.07		^200^
ITO/ZnO:ETP	MoO_3_/Ag	PBDB-T-2F:IT-4F	0.85	20.14	74.4	12.9		^200^
ZnO/Ag(O)/ZnO	PEDOT:PSS/Ag	PTB7-Th:PC71BM	0.77	17.98	58.4	8.04	Fully flexible solar cell on top of PET substrate. ITO free	^201^
ITO/Ga–ZnO NPs	MoO_3_/Au	P3HT:PCBM	0.42	11.7	39.7	1.95		^202^
ITO/Li–ZnO NPs	MoO_3_/Al	P3HT:PCBM	0.61	9.93	68	4.07		^205^
FTO/La–ZnO NPs	V_2_O_5_/Ag	P3HT:PCBM	0.63	11.65	59	4.10		^204^
ITO/Hf–In–ZnO	V_2_O_5_/Ag	PTB7:PC70BM	0.67	16.35	37.8	4.77		^206^
ITO/ZnO	MoO_3_/Al	PBDB-T:ITIC	0.90	17.2	73	10.7		^209^
ITO/EDTA:ZnO	MoO_3_/Al	PBDB-T:IT-M	0.95	17.06	72.1	11.7	Annealed at low temperature of 150 °C	^210^
ITO/ZnO:HO-PBI	MoO_3_/Al	PDBD-T-2F:Y6	0.83	25.34	74.8	15.7		^211^

Very recently, however, it has been known that the fullerene-based OSCs' performances are being limited by its poor light absorption, as well as instability in morphology.^212^ Huge efforts are made to substitute PCBM and the other fullerene-derived acceptor with a non-fullerene one. Zhao *et al.* demonstrated the usage of ITIC, a non-fullerene n-type acceptor, coupled with PBDB-T to create an active layer, with ZnO as a buffer layer. ZnO in this demonstration was synthesized *via* precursor solution method, and the resulting inverted cell exhibited a PCE of 10.71%, as well as excellent stability of 83% retained PCE after over 4000 h.^209^ However, further annealing at high temperature was needed to reduce the defects, which is energy consuming and not desirable, especially when working with a polymeric substrate. Later in 2017, Li *et al.* incorporated ethylene diamine tetraacetic acid (EDTA) with the ZnO precursor to passivize the defects in ZnO due to its chelation function, effectively lowering the required annealing temperature. On the other hand, the low conductivity of EDTA can be mitigated by ZnO's high conductivity. The combination of the two, in conjunction with PBDB-T:IT-M BHJ active layer solar cell, displays a PCE of 11.67%, and a FF of 72.1%.^210^ The previously mentioned solar cell employing ETP as down-conversion material was also tested using this approach, replacing the PTB7-Th:PC71BM with PBDB-T-2F:IT-4F, increasing its efficiency from 9.22% to 13.12%.^200^ Another non-fullerene active layer, PDBD-T-2F:Y6, has been utilized as a BHJ active layer, with the ZnO thin film as the buffer layer. This time, however, the ligand was also used in the form of tetrahydroxy-perylene bismide (HO-PBI ligand), embedded onto a ZnO thin film, improving the device efficiency up to 15.73%, making it one of the highest reported non-fullerene based OSCs to date.^211^ The high efficiency is attributed to the higher solubility and better molecular dispersion of ZnO:HO-PBI, resulting in the robust coordination between organic molecules and the metal oxide lattice, leading to increased electron mobility and easier electron transport.

## 3 Summary and future outlook

In this review, the application of ZnO as an active material in emerging solar cells technologies, including dye-sensitized solar cell (DSSC), QDSC (ouantum-dots sensitized solar cell), PSC (perovskite-sensitized solar cell), inorganic solar cell, Organic Solar Cell (OSC), Hybrid Solar Cell (HSC) is discussed. The inorganic solar cell is one of the earliest generations of solar cells, and is more mature than polymeric-based devices. Various combinations of metals, alloys and oxides, as well as unique nanostructures, fabrication methods, elemental doping and interfacial modifications, have been studied to increase the cell performances and stability. However, the issues regarding the abundance and toxicity must be addressed because many of the highest performing cells contain toxic elements, such as Cd and Pb. At the same time, the ‘safer’ alternatives found themselves unable to compete with the performances of the next-generation solar cells. Although many have shifted to a ‘newer’ generation of solar cell architectures, including organic, perovskite, and sensitized cells, the fully inorganic solar cells are still massively explored and experimented. Optimizations in various aspects can always be done, including the nanostructure dimension and spacing in the vertically aligned nanostructures, suitable doping, post-and treatment processes. With proper adjustments, the fully inorganic solar cells have the potential to be a promising candidate for a low-cost, reproducible photovoltaic device.

To date, the blend of PCBM and P3HT remains the most widely used blend of an active layer in OSCs. However, the combination of the two is not necessarily the best for achieving maximum efficiency, particularly due to the small energy difference between the PCBM's LUMO and P3HT's HOMO. As discussed in this review, a change in the material that can enlarge the difference between the two energies is necessary to improve the efficiency, which has been demonstrated by substituting PCBM with ICBA, or even with other non-fullerene derivatives. Optimization of the ZnO CBL, ranging from the favourable morphology of the 1D nanostructure, to details such as the nanorod/nanowire length, is also an important factor to determine the final performance. Nevertheless, many agreed that the utilization of ZnO CBL is one of the most promising ways to achieve more efficient, more stable, and more reproducible OSCs, due to its favourable energy levels, possibility to provide an ohmic contact, excellent stability, as well as ease of synthesis and low cost. OSCs made by incorporating ZnO as CBL, particularly the ones with inverted structure, is quickly becoming a very promising solution to fabricate a low-cost, high performance and high stability photovoltaic device.

So far, reports of ZnO/organic HSCs performances are still lower than those of OSCs, especially against the PCBM/polymer based OSCs. Although the inorganic phase has been theorized to exhibit higher electron mobility, in practice, it does not always translate to higher performance due to several other factors mentioned before. Lower current densities are usually found across HSCs when compared to OSCs due to the poor polymer infiltration in the nanostructure, low polymer/ZnO wettability, and the small interfacial area between donor and acceptor. Low charge carrier mobility is also observed in HSCs utilizing P3HT as the organic component. P3HT was used due to its exceptional performance in PCBM/P3HT OSCs. However, the morphology of ZnO in the active layer may confine the P3HT, which may lead to much lower hole mobility, resulting in low PCE of the HSCs. Similar findings were also reported with MDMO-PPV as an organic semiconductor. Thus, it may be another topic of interest to couple ZnO nanostructures with another less explored p-type organic semiconductors. In the specific case of randomly dispersed nanocrystal solar cell architecture, the random dispersion of ZnO nanocrystals may be attributed to the reduced current density due to the unavailability of a direct electron transport pathway. Additional problems may arise when utilizing pre-synthesized nc-ZnO, in which solvent evaporation during synthesis may cause ZnO nanocrystals to agglomerate, reducing the current density even further down. The bilayer structure suffers mainly from a small interfacial area between the two phases. Although theoretically, the vertically aligned structures are the most ideal configuration for this application due to the structure's ability to provide a direct pathway for charge carrier collection, reports regarding this particular architecture so far have been relatively modest. This is particularly due to the low polymer infiltration and crystallinity, which can be relatively low even after thermal annealing, as well as unoptimized properties, such as unfavourable nanostructure morphologies. However, with proper morphology control (*e.g.*, the spacing between nanorods should match the exciton diffusion length of the polymer, optimized nanorod/nanowire length), as well as the optimized blocking layer thickness, it is believed that cells made with this architecture can still be optimized and improved immensely in the future.

**Fig. 3:**
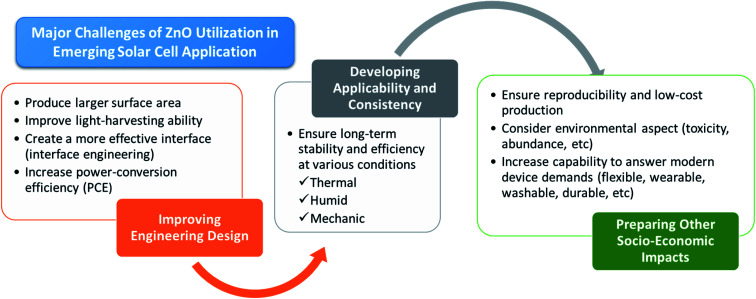
Major challenges for ZnO solar cell development.

Despite many signs of progress achieved so far in terms of ZnO development, there is still plenty of room for improvement. Fig. 3 depicts the major challenges that need to be overcome for ZnO to be superior in solar cell applications. A higher surface area and light-harvesting ability are among the critical properties that need to be improved. In terms of the solar cell systems, the interface should be more effective than the current progress. Overall, these improvements would lead to a higher power-conversion efficiency and longer lifetime in various environments with a different thermal condition, humidity, and mechanical loading.

In general, emerging solar cell technologies are considered to have low penetration into the market. Thus, progress in achieving a low-cost technique to prepare ZnO with good reproducibility alongside a green method that provides sustainability and environmental-friendliness will boost the positive impacts on the socio-economic aspects. Besides, the capability of the whole device to be easily adjusted for various device requirements (flexible, wearable, durable, washable, *etc.*) is also vital for enhancing the commercialization.

## Conflicts of interest

There are no conflicts to declare.

## Supplementary Material
